# A tale of two stories: COVID-19 and disability. A critical scoping review of the literature on the effects of the pandemic among athletes with disabilities and para-athletes

**DOI:** 10.3389/fphys.2022.967661

**Published:** 2022-11-09

**Authors:** Luca Puce, Khaled Trabelsi, Achraf Ammar, Georges Jabbour, Lucio Marinelli, Laura Mori, Jude Dzevela Kong, Christina Tsigalou, Filippo Cotellessa, Cristina Schenone, Mohammad Hossein Samanipour, Carlo Biz, Pietro Ruggieri, Carlo Trompetto, Nicola Luigi Bragazzi

**Affiliations:** ^1^ DINOGMI, University of Genoa, Genoa, Italy; ^2^ Institut Supérieur Du Sport et de L’Éducation Physique de Sfax, Université de Sfax, Sfax, Tunisia; ^3^ Research Laboratory: Education, Motricité, Sport et Santé, EM2S, LR19JS01, Sfax University, Sfax, Tunisia; ^4^ Department of Training and Movement Science, Institute of Sport Science, Johannes Gutenberg-University Mainz, Mainz, Germany; ^5^ Laboratoire Interdisciplinaire en Neurosciences, Physiologie et Psychologie: Activité Physique, Santé et Apprentissages (LINP2-APSA), UFR STAPS, UPL, Université Paris Nanterre, Nanterre, France; ^6^ Physical Education Department, College of Education, Qatar University, Doha, Qatar; ^7^ Istituto di Ricovero e Cura a Carattere Scientifico (IRCCS) Ospedale Policlinico San Martino, Genoa, Italy; ^8^ Laboratory for Industrial and Applied Mathematics (LIAM), Department of Mathematics and Statistics, York University, Toronto, ON, Canada; ^9^ Laboratory of Microbiology, Department of Medicine, Democritus University of Thrace, Dragana, Greece; ^10^ Department of Sport Science, Imam Khomeini International University, Qazvin, Iran; ^11^ Orthopedics and Orthopedic Oncology, Department of Surgery, Oncology and Gastroenterology (DiSCOG), University of Padova, Padova, Italy

**Keywords:** COVID-19, para athletes, disability in sport, scoping review, critical review, research methodology

## Abstract

The still ongoing COVID-19 pandemic has dramatically impacted athletes, and, in particular, para-athletes and athletes with disabilities. However, there is no scholarly appraisal on this topic. Therefore, a critical scoping review of the literature was conducted. We were able to retrieve sixteen relevant studies. The sample size ranged from 4 to 183. Most studies were observational, cross-sectional, and questionnaire-based surveys, two studies were interventional, and two were longitudinal. One study was a technical feasibility study. Almost all studies were conducted as single-country studies, with the exception of one multi-country investigation. Five major topics/themes could be identified: namely, 1) impact of COVID-19-induced confinement on training and lifestyles in athletes with disabilities/para-athletes; 2) impact of COVID-19-induced confinement on mental health in athletes with disabilities/para-athletes; 3) impact of COVID-19-induced confinement on performance outcomes in athletes with disabilities/para-athletes; 4) risk of contracting COVID-19 among athletes with disabilities/para-athletes; and, finally, 5) impact of COVID-19 infection on athletes with disabilities/para-athletes. The scholarly literature assessed was highly heterogeneous, with contrasting findings, and various methodological limitations. Based on our considerations, we recommend that standardized, reliable tools should be utilized and new, specific questionnaires should be created, tested for reliability, and validated. High-quality, multi-center, cross-countries, longitudinal surveys should be conducted to overcome current shortcomings. Involving all relevant actors and stakeholders, including various national and international Paralympic Committees, as a few studies have done, is fundamental: community-led, participatory research can help identify gaps in the current knowledge about sports-related practices among the population of athletes with disabilities during an unprecedented period of measures undertaken that have significantly affected everyday life. Moreover, this could advance the field, by capturing the needs of para-athletes and athletes with disabilities and enabling the design of a truly “disability-inclusive response” to COVID-19 and similar future conditions/situations. Furthermore, follow-up studies on COVID-19-infected para-athletes and athletes with disabilities should be conducted. Evidence of long-term effects of COVID-19 is available only for able-bodied athletes, for whom cardiorespiratory residual alterations and mental health issues a long time after COVID-19 have been described.

## Introduction

The still ongoing “Coronavirus Disease 2019” (COVID-19) outbreak, caused by an emerging infectious agent, termed “Severe Acute Respiratory Syndrome Coronavirus type 2” (SARS-CoV-2) (Sharma et al., 2021), has been affecting more than 200 countries since early 2020. Initially declared by the World Health Organization (WHO) a “Public Health Emergency of International Concern” (PHEIC), at the end of January 2020, and, subsequently, a pandemic, it has significantly overwhelmed healthcare infrastructure worldwide (Singhal, 2020; Bernacki et al., 2021). Given the initial lack of availability of effective drugs and vaccines, in order to control and contain the pandemic, governments and authorities have had to implement a package of public health interventions, such as social/physical distancing, hygiene/sanitation protocols (hand washing, use of masks and other personal protective equipment, PPE, devices), and self-isolation (safer-at-home, stay-at-home, shelter-in-place, quarantine, and even lockdown), collectively known as non-pharmaceutical interventions (NPIs), the stringency of which has significantly varied across the world (Perra, 2021). In some countries (such as Italy), NPIs have included the ban of any kind of inter-household mingling and/or outdoor activities, including sports and physical activity. In contrast, in other countries (like the United Kingdom, Germany, or Poland), certain types of activities were allowed. Other countries, like Sweden, have, instead, avoided the implementation of stringent protocols and have only recommended precautionary measures (Seale et al., 2020; Urbański et al., 2021).

All this has dramatically impacted the physical level of entire populations, especially affecting socially vulnerable communities, such as populations with disabilities (Jesus et al., 2020; Jesus et al., 2021a; Jesus et al., 2021b; Kamalakannan et al., 2021), who presented greater infection risks due to multiple, intersecting mediators (i.e., lack of accessible evidence-based information, structural barriers, difficulties in complying with COVID-19 induced restrictions, institutional ableism in health care and unethical disadvantages in the rationing of the delivery of lifesaving and critical care provisions), as well as psychological distress and trauma (due to the fear of COVID-19, isolation, loneliness, deaths and illnesses of loved ones and community members, retaliation, and interpersonal violence, among others) (Jesus et al., 2020; Lund, 2020; Lund et al., 2020; Jesus et al., 2021a; Jesus et al., 2021b; Kamalakannan et al., 2021; Goddard et al., 2022).

On the one hand, physical activity and exercise could counteract or, at least partially, mitigate against this burden (Chtourou et al., 2020; Ghram et al., 2021), especially in people living with disabilities. An accumulating body of evidence has, indeed, shown that regularly practicing sports can promote rehabilitation, and improve physical and mental health and wellbeing, resulting in better self-confidence, self-esteem, psychological balance, self-acceptance, social inclusion, and integration, as well as enhanced quality of life (Puce et al., 2017; Puce et al., 2019; Brancher et al., 2021). On the other hand, COVID-19 has affected the sports world as well, disrupting the training and preparation of athletes, causing, in some cases, reduced opportunities to access gyms, fitness centers, and other sports infrastructure, and resulting in confinement-induced detraining in several cardio-pulmonary (i.e., cardiac output, VO_2max_, maximal stroke volume, and artero-venous O_2_ difference) and musculoskeletal (muscle mass, strength, and power) variables (Cavaggioni et al., 2022). Moreover, access to high-quality food, which is of paramount importance for elite athletes, has been particularly challenging, with many athletes experiencing “food insecurity” (Roberts et al., 2020; Abbey et al., 2022). Furthermore, athletes have had to cope with an unprecedented situation, characterized by the loss of daily routine (consisting of scheduled training appointments, and matches), and the postponement or even cancellation of major national and international sports events (like national championships and the Tokyo 2020 Olympic and Paralympic Games), with subsequent career and financial insecurity (Håkansson et al., 2020; Dehghansai et al., 2021; Busch et al., 2022). All this uncertainty, along with the necessity to set new goals, has contributed to psychological distress and feelings of depression and anxiety (Busch et al., 2022).

If the impact of COVID-19 on athletes has been investigated (Clemente-Suárez et al., 2020; Tomovic and Krzman, 2020; Harangi-Rákos et al., 2022; Romdhani et al., 2022), there is a dearth of data concerning the effects of the pandemic on para-athletes and athletes with disabilities. Therefore, to fill in this gap in knowledge, we carried out the present scoping review.

## Material and methods

### Study design choice and strategy

Given the breadth (rather than the width) of our research questions and the complex, intersectional nature of our study (situated at the intersection of sport, disability, and COVID-19), we opted for a scoping review (rather than for a systematic review). A scoping review represents an emerging technique for synthesizing the existing scholarly literature on a given topic (Moher et al., 2015; Khalil and Tricco, 2022; Munn et al., 2022). Scoping studies or scoping reviews “aim to map rapidly the key concepts underpinning a research area and the main sources and types of evidence available, and can be undertaken as stand-alone projects in their own right, especially where an area is complex or has not been reviewed comprehensively before” (Arksey and O’Malley, 2005). We leveraged Arksey and O'Malley (2005), Levac et al. (2010), Colquhoun et al. (2014), Peters et al. (2015), and Tricco et al. (2016) working definitions and frameworks of scoping reviews. In particular, Arksey and O’Malley (2005) recommend a six-stage approach. The steps and the iterative processes followed and implemented in the present scoping study are briefly overviewed in the following sub-sections.

Stage 1: Identification of the research question(s) and team building and development

The “population/participants-concepts-context” (PCC) mnemonic was utilized to generate a preliminary search strategy, ensuring both breadth and relevance of coverage, as recommended by the Joanna Briggs Institute for scoping reviews (2015). “Population/participants” were para-athletes and athletes with disabilities, the main “concept” was the impact of COVID-19 on this specific population; and, the “context” was worldwide (we did not restrain our search to a particular territory/geographic location).

More precisely, concerning the population of our study, we note that terms, such as “adapted sport,” “disability sport,” “Paralympic sport,” and “Para sport,” are words commonly used in an interchangeable fashion to indicate sports disciplines that accommodate people with any kind of disability, including physical/motor, sensory and intellectual/relational/developmental disabilities, practicing sports as a recreational physical activity or participating in organized competitions (Brancher et al., 2021). On the other hand, “Paralympic sport” is often utilized as a term to specifically describe sports disciplines that compete in Paralympic events, such as the Paralympic Games (Brittain, 2016; Townsend et al., 2018), organized under the auspices of the “International Paralympic Committee” (IPC), the global governing body responsible for the development of Para sport and, more generally speaking, a more socially just and inclusive sports world.

After rapidly scanning and familiarizing ourselves with the existing scholarly literature, we were able to specify and formulate our research questions: “What is the impact of the still ongoing COVID-19 pandemic on para-athletes and athletes with disabilities, in terms of health risks of contracting the virus and developing the infection, and disruptive effects, if any, posed by COVID-19 on training and performance outcomes, physical and mental health and well-being? Which strategies can be adopted and implemented to protect para-athletes and athletes with disabilities against the health risks imposed by COVID-19? Which interventions can be applied to counteract/minimize the effects of detraining and optimize training protocols and conditioning programs among para-athletes and athletes with disabilities?”.

Then, we assembled our interdisciplinary team. Given the complex, intersectional nature of the topic and the different competencies and skills required, our team consisted of specialists from different disciplines: public and global health, epidemiology, biostatistics and research methodology, infectious diseases, microbiology, and, in particular, virology, sports sciences, orthopedics, sports and exercise medicine, rehabilitation medicine, and physical therapy.

Subsequently, a team leader was identified and formally designated. Given *their* multidisciplinary skills, NLB were indicated as the team coordinator: NLB are a queer, gender-diverse scientist and medical doctor, with a specialization in public health, epidemiology, and biostatistics, and are strongly passionate about equity, diversity, and inclusion (EDI) principles. NLB also act as the main contact author/corresponding author of the present study.

We devised an *a priori* protocol, as recommended. This can be accessed upon written request to the corresponding author.

Stage 2: Identification of relevant literature and literature search strategy

Two major scholarly electronic databases were consulted: namely, MEDLINE (accessed *via* PubMed’s freely available interface) and Scopus (Elsevier). They were mined since the inception of the infectious outbreak, with no language restrictions. The search string consisted of two major components: para-athletes/athletes with disabilities and COVID-19, with synonyms/variants properly linked by using Boolean operators. “Medical subject headings” (MeSH) terms and wild-card (truncated words) options were used when appropriate. The full search string is reported in Table 1. Extensive cross-referencing was applied: reference lists of eligible studies were scanned for getting further relevant articles not returned by the keyword-based search. This was done iteratively until “search saturation”, that is to say, no new relevant articles could be found. Existing reviews on similar or related topics, if existing (Alcaraz-Rodríguez et al., 2021), were scanned to increase the chance of including all relevant studies. Further, specific target journals were hand-searched for relevant studies. Finally, also gray literature was consulted, by mining the “Directory of Open Access Journals” (DOAJ) and Google Scholar.

Stage 3: Selection of the studies

**TABLE 1:** Search strategy adopted in the present review.

Search strategy item	Details
Databases searched	PubMed/MEDLINE, Scopus
Keywords	(SARS-CoV-2 OR COVID-19 OR “novel emerging coronavirus”) AND (“disabled athletes” OR “athletes with disabilities” OR “para-athletes” OR “paralympic” OR “wheelchair sport” OR “amputee athletes” OR “athletes with limb deficiency” OR “athletes with visual impairment” OR “athletes with sensory impairment” OR “athletes with motor impairment” OR “athletes with cerebral palsy” OR “athletes with spinal cord injury” OR “disabled players” OR “players with disabilities” OR “players with cerebral palsy”)
Inclusion criteria	P: athletes with disabilities/para-athletes E: potentially exposed to the novel emerging coronavirus C: among different para-sport disciplines; against the general population O: the risk of contracting the virus; the impact of the pandemic on training, mental health S: any study design (quantitative, qualitative, retrospective, prospective; cross-sectional, longitudinal; observational, interventional)
Exclusion criteria	P: people with disabilities practicing sports and physical activities E: non-potentially exposed to SARS-CoV-2 O: any other outcome
Time filter	Since the inception of the infectious outbreak
Language restriction	None applied
Hand-searched target journals	Disabil Health J; Front Sports Act Living; Int J Environ Res Public Health; J Sports Med Phys Fitness

Studies were selected for inclusion based on pre-specified inclusion and exclusion criteria, which were devised and formulated based both on the PCC mnemonic and the “population/participants-intervention-comparator/comparison-outcome-study design” (PICOS)/“population/participants-exposure-comparator/comparison-outcome-study design” (PECOS) components.

Studies were included if focusing on a population of para-athletes/athletes with disabilities (P), potentially exposed to the novel emerging coronavirus (E), or observing the COVID-19 restrictions (practicing social/physical distancing, quarantine, wearing masks, etc.) and/or undergoing personalized, adapted/modified training protocols during the pandemic (I). Studies could be included if comparing different para-sport disciplines; against the general population, and/or able-bodied athletes. Other comparisons of interest included sex/gender- and age-specific comparisons (C). Outcomes of interest were the quantification of the risk of contracting the virus; the rate of infection among para-athletes/athletes with disabilities; the impact of the pandemic on lifestyles, training, and performance outcomes, and physical and mental health/well-being (O). Any study design was eligible for inclusion: retrospective, prospective, questionnaire- or test-based, quantitative, qualitative, or mixed-method studies (S).

Stage 4: Mapping out the data, data charting, and extraction

An Excel spreadsheet contained information on how and which relevant data to extract. Then, we utilized the “Research Electronic Data Capture” (REDCap) software to store the retrieved articles (both deemed eligible for inclusion and those finally included in the present scoping review) and assist/facilitate data extraction. REDCap is a secure, web-based platform that efficiently supports and enhances data capture for research purposes (Westphaln et al., 2021). Moreover, since our team is highly multidisciplinary and diverse, REDCap allows the inclusion of multiple users within projects. Further, it can accommodate multiple types of data, which, in our case, were rather heterogeneous. It provides audit trails and quality checks for tracking and monitoring data manipulation and exportation. Also, REDCap supports and facilitates data exportation into many types of statistical software for their further processing and analysis (Westphaln et al., 2021). These functions allow REDCap to properly accommodate all the processes of full-text review and data extraction/manipulation for large and diverse research teams.

Data extraction quality was checked by computing the pooled kappa statistics as a quantitative measure of the inter-rater reliability/agreement, as recommended by Tricco et al. (2015).

Stage 5: Data collating, summary, synthesis, and reporting

Data extracted were tabulated and synthesized in a narrative fashion. Major topics/themes were identified by means of thematic analysis and overviewed qualitatively. Furthermore, we followed the “Preferred Reporting Items for Systematic Reviews and Meta-Analyses” (PRISMA) extension for scoping reviews (PRISMA-Scr) (Tricco et al., 2018).

Stage 6: Expert consultation/“consultation exercise”

Our team identified experts on the topic of sports and disability, as well as on the topic of COVID-19. For this, we used cues from the identified literature, as well as cues from electronic databases (such as Scopus) which enable the visualization of a list of the top-cited and most productive scholars on a given topic. Some of these experts were consulted during the preparation of the manuscript to provide critical feedback on our work.

### Quality assessment

A thorough, formal quality appraisal was not conducted given that is not a mandatory component of scoping reviews, while an informal assessment was carried out, by noting down the methodological limitations of each study and providing an overall qualitative synthesis of the shortcomings. This rapid, informal assessment was instrumental to the recommendations we were able to formulate.

## Results

### Outcomes of the literature search strategy

The initial literature search yielded a pool of 123 items, by searching PubMed/MEDLINE and Scopus. After removing duplicates, seventy items were examined and 54 items were further discarded based on title and/or abstract. Sixteen studies were considered potentially eligible and were read in full text. Two studies were subsequently discarded with reason: one because it did not contain any original data concerning para-athletes (Håkansson et al., 2020), and one because the population did not meet inclusion criteria (people with disabilities, but not athletes with disabilities) (Kamyuka et al., 2020). Subsequently, fourteen articles were retained in the present scoping review. Two further studies were retrieved by searching Google Scholar. The final number of included studies was sixteen. Their major characteristics and findings are summarized in Table 2, to which the reader is referred.

**TABLE 2:** Main features of included studies. Abbreviations: BFPI, Big Five Personality Inventory; CAS, coronavirus anxiety; CMJ, countermovement jump; FFQ, Food Frequency Questionnaire; MAT, modified agility *t*-test; NA, not available; 1RM, one repetition maximum; PHQ-4, Patient Health Questionnaire 4; PSS, Perceived Stress Scale; RPE, rating of perceived exertion; STAI, State-Trait Anxiety Inventory; TSS, Training Stress Score.

References	Study design	Country/countries	Study period	Sample size, sex/gender, and mean age	Para-sport	Measures	Main findings
Busch et al. (2022)	Longitudinal study	Germany	March 2020-April 2021: eight time points (March 27 to 6 April 2020, April 24 to 4 May 2020, May 15 to 25 May 2020, June 5 to 15 June 2020, September 25 to 5 October 2020, October 23 to 2 November 2020, January 1 to 11 January 2021, and March 26 to 5 April 2021)	78; 40 F, 38 M; 29.8 ± 11.4 years	Paralympic athletes	PHQ-4	↓ PHQ-4 scores
Cavaggioni et al. (2022)	Longitudinal case series/interventional study with a pre-post assessment	Italy	Three time points: end of February 2020 (2 weeks prior to COVID-19 lockdown), March 2020-September 2020, and 16 weeks after returning to a regular gym-training	4; 1 F, 3 M; 24.3 ± 3.8 years	Para swimmers	Upper-body muscular strength and power—1RM, mean propulsive velocity of the barbell, mean propulsive velocity close to 1RM, and mean relative propulsive power (lat pull-down and bench press exercises)	↓ muscular strength and power
Clemente-Suárez et al. (2020)	Online questionnaire-based study	Spain	From March 24 to 12 April 2020	39 (vs*.* 175 Olympic athletes); 31.8 ± 9.3 years	Paralympic athletes; athletics n = 8, basketball n = 6, canoe n = 1, cycling n = 4, football n = 1, judo n = 1, swimming n = 9, table tennis n = 5, taekwondo n = 1, triathlon n = 2, weightlifting n = 1	Individual perceptions about the COVID-19 crisis; psychological profile (BFPI, UCLA Loneliness Scale, Acceptance and Action Questionnaire-II, STAI)	↑ importance placed on the confinement ↑ perceived support from public institutions ↑ conscientiousness
Denerel and Lima, (2022)	Online questionnaire-based study	Turkey	Between 15 June 2021 and 15 July 2021	362 (183 with disabilities)	Athletes with disabilities	Depression, anxiety, stress, CAS, and nonspecific psychological distress	↑ higher depression, anxiety, and CAS scores
Fiorilli et al. (2021)	Questionnaire-based, case-control study	Italy	From 12 March 2020, to 3 May 2020	146 (73 with disabilities, 73 able-bodied); 42.11 ± 13.70 years (with disabilities), 40.23 ± 13.73 years (able-bodied)	Athletes with disabilities (blind, 52%, with mobility impairment, 34%, such as limb amputation, and deaf, 14%)	Psychological distress	↓ subjective distress
Hu et al. (2021)	Qualitative study (in-depth, semistructured interviews)	United States	NA	29; 18 F, 11 M; 18–62 years	Thirteen different Paralympic sports (boccia, cycling, equestrian, fencing, goalball, paracanoeing, powerlifting, rugby, sitting volleyball, swimming, taekwondo, wheelchair racer, and wheelchair tennis)	Athletic identity (Disability Sport Athletic Identity)	↑ multiplicity of roles ↑ mental adaptation
Kaneda et al. (2021)	Questionnaire-based study	Japan	From 16 April to 24 May 2020	39 (20 with disabilities; 19 able-bodied); 8 F, 11 M	Para swimmers	Mental health and apathy	↑ mental health ↑ apathy
Kaneda et al. (2022)	Databased-based epidemiological survey	Japan	From August 17 to September 5 and from July 13 to August 8	NA	Paralympic athletes (*versus* Olympic athletes)	COVID-19 infection rate	↑ than able-bodied athletes
Kubosch et al. (2021)	Cross-sectional, questionnaire-based study	Germany	From May 17 to 30 August 2020	109; 57 F, 52 M; 29.2 ± 10.4 years	Paralympic athletes	Well-being Satisfaction with training Financial preoccupations	↓ well-being ↓ satisfaction with training ↑ financial preoccupations
Martínez-Patiño et al. (2021)	Questionnaire-based study	Spain	Recruitment from 2 to 22 September 2020	511 (64 with disabilities, 447 able-bodied); NA; 28.44 ± 10.50 years	Paralympic athletes from athletics, basketball, cycling, weightlifting, horse riding, judo, swimming, canoeing, taekwondo, tennis, table tennis, archery, Olympic shooting, and triathlon	Perception of threat, stress (PSS-10), state of mind, and training patterns	↑ coping with personal problems ↓ feelings of being lonely ↑ approval of confinement
Muti et al. (2022)	Technical feasibility study	Italy (Piediluco, Umbria, at the Center for preparation of the Olympic and Paralympic National Italian Rowing team; Varese, and Gavirate)	During each retreat of the Italian Paralympic rowing team and during National and International rowing competitions Each retreat lasted 7–14 days; for a total of 11 retreats Duration of the study 10 months, from November 2020 to August 2021	23 (16 athletes, 5 coaches, and 2 staff members; 12 at the follow-up); 9 F, 7 M; 18–55 years (35.38 ± 12.88 years)	Rowing para-athletes	Risk of contracting the coronavirus	No clusters of coronavirus
Peña-González et al. (2022)	Interventional study with a pre-post assessment	Spain	Testing session before commencement of the lockdown and second timepoint 12 weeks after	15; 15 M; 18 [21–30] years	Football players with cerebral palsy	Fitness (free CMJ, 5-, 10-, 20-m sprint, MAT, dribbling test)	= fitness (↑ CMJ)
Schipman et al. (2022)	Case-control study	Germany	255 Paralympic events	NA	Paralympic athletes taking part in 255 Paralympic events since 2010	Performance outcomes	↓ performance outcomes
Shaw et al. (2021)	Questionnaire-based retrospective, pre-post study	Canada, United Kingdom, Australia, South Africa, Belgium	Recruitment during May-mid June 2020; four time-points (February, March, April, and May)	25; 15 F, 10 M; 21–60 years	Para-cyclists and para triathletes	Training (TSS) Caloric intake (FFQ) Fitness (ramp exercise test) Time spent engaging in sedentary screen time activities	= training volume and intensity = caloric intake = fitness (↑ duration time, = average power, = maximum power, = average heart rate, = max heart rate) ↑ sedentary behavior
Trigo et al. (2022)	Cross-sectional, questionnaire-based study	Brazil	May 2020	180; 72 F, 107 M; 33.85 ± 9.41 years	Athletes from 22 different para-sports with several types of impairment (visual impairment, intellectual impairment, limb deficiency, short stature, leg length difference, impaired muscle power, impaired passive range of movement, coordination impairment—hypertonia, ataxia, and athetosis). Para sports were divided into Individual sports (athletics, boccia, cycling, powerlifting, rowing, swimming, triathlon); individual sports with opposition (badminton, judo, Table tennis, taekwondo, wheelchair fencing, and wheelchair tennis); and team sports (football 5-a-side, goalball, sitting volleyball, wheelchair basketball, wheelchair rugby)	Hours of training, number of sessions, and RPE	↓ hours of training, number of sessions, and RPE = life satisfaction ↓ perceptions on the impact of COVID-19 on performance outcomes and career
Urbański et al. (2021)	Cross-sectional questionnaire-based study	Poland	Recruitment from May to June 2020 Questionnaire period from May 20 to 30 April 2020	166; 66 F, 100 M; 33 ± 11.7 years	Athletes with disabilities (spinal cord injury and limb amputation/deficiency) from 15 different sports disciplines	Access to sports infrastructures and weekly training time Satisfaction with training	↓ access to infrastructure ↓ weekly training time ↓ satisfaction with training

### Features of studies included

The sample size ranged from 4 to 183. Most studies were observational, cross-sectional, and questionnaire-based surveys, two studies were interventional (Cavaggioni et al., 2022; Peña-González et al., 2022), two were longitudinal (Busch et al., 2022; Cavaggioni et al., 2022). One study was a technical feasibility study (Muti et al., 2022). Almost all studies were conducted as single-country studies, with the exception of the multi-country investigation by Shaw et al. (2021). Five major topics/themes could be identified: namely, 1) impact of COVID-19-induced confinement on training and lifestyles in para-athletes/athletes with disabilities; 2) impact of COVID-19-induced confinement on mental health in para-athletes/athletes with disabilities; 3) impact of COVID-19-induced confinement on performance outcomes in para-athletes/athletes with disabilities; 4) risk of contracting COVID-19 among para-athletes/athletes with disabilities; and, finally, 5) impact of COVID-19 infection on para-athletes/athletes with disabilities.

### Impact of COVID-19-induced confinement on training and lifestyles in para-athletes/athletes with disabilities

Six studies (Shaw et al., 2021; Urbański et al., 2021; Cavaggioni et al., 2022; Peña-González et al., 2022; Schipman et al., 2022; Trigo et al., 2022) have assessed the impact of COVID-19 on training and lifestyles in para-athletes/athletes with disabilities. Shaw et al. (2021) conducted a survey concerning the impact of the pandemic on diet, fitness, and sedentary behaviors in a sample of 25 elite national and international (from Canada, the United Kinggdom, Australia, United States, South Africa, and Belgium) para cyclists and para triathletes recruited in the period from early May to mid-June 2020.15 were females, and 10 were males. Age ranged from 21 to 60 years. Ramp exercise test duration varied significantly between pre- (27.3 ± 5.8 min) and post-pandemic (28.0 ± 6.4), suggesting an improving trend, but no effects in terms of average and maximum power, as well as average and maximum heart rate, could be found. The authors concluded that, overall, COVID-19 did not impact the training volume or intensity or the fitness of para cyclists, also when comparing data between February and May 2020 (where the motivation levels and other psychological parameters were expected to be low). Sex- and gender-specific differences could be computed in dietary uptake concerning several parameters and macro- and micro-nutrients (namely, energy, carbohydrates, sugar, protein, total fat, saturated, monounsaturated, and polyunsaturated fat, folate, iron, potassium, sodium, vitamins B1, B12, B3, B6, and D, and zinc), with lower intakes in females. However, dietary uptake did not differ between pre- and post-pandemic. Furthermore, the authors found that, whereas no temporal differences could be detected concerning energy intake and exercise expenditure, the latter parameter could be decreased, due to an increase in sedentary screen time (from 4.5 ± 1.9 h per day to 6.1 ± 1.5 h per day, *p* = 0.001), leading to a net caloric imbalance, which, however, is unlikely to have any consequences in a sample of highly trained, elite athletes and, being short-lived, may quickly restore, returning to the baseline levels prior to the pandemic, once COVID-19 induced strictures are lifted.

Similarly, Peña-González et al. (2022), performing a 12-week interventional study in an ecological environment with a pre-post assessment, found that international football players with cerebral palsy were able to maintain adequate levels of self-training (which consisted of combined strength, and resistance exercises, four training sessions per week) during the COVID-19 pandemic, thus preserving their fitness. An improvement in free countermovement jump (CMJ) was computed (from 28.60 ± 6.18 cm to 32.79 ± 6.69 cm, *p*-value <0.001, with an increase of 4.19 [95%CI 2.46–5.93] cm), whereas the other fitness variables (5-m, 10-m, and 20-m sprints, the modified agility *t*-test or MAT, and the dribbling test) remained stable. Differences between sports classes (severe impairment or FT1, moderate impairment or FT2, and mild impairment or FT3) could not be found. Finally, the authors speculated that the increase in free CMJ could be due to the fact that in the modified training protocol there was an over-representation of vertical, rather than horizontal, exercises.

Contrasting findings were, instead, obtained by Cavaggioni et al. (2022), who retrospectively analyzed a case series consisting of four para swimmers. One had cerebral palsy and belonged to the S5 para-swimming functional class, one had a hereditary spastic paraparesis (S6 class), one had a lower limb deficiency (S9 class), and one had lower limb amputation (S8 class). The authors evaluated the effectiveness of muscular strength and power, dry-land home training, and found a very likely/substantial decrement in one-repetition maximum, mean propulsive velocity, and mean relative propulsive power during the lockdown period, which was reverted when COVID-19-induced restrictions were lifted and para-athletes could return to a regular gym-training program. The decrease in neuromuscular variables (including motor unit recruitment, and firing rate) was potentially due to the fact that equipment available at home (weights, and elastic band), while, on the one hand, ensuring a certain degree of continuity in training, was not enough, on the other hand, to enable the execution of moderate-to high-intensity resistance exercises.

In line with Cavaggioni et al. (2022), Trigo et al. (2022), in a cross-sectional, questionnaire-based survey in Brazil, obtained comparable results. The authors had recruited participants with 9.83 ± 5.73 years and 7.49 ± 5.26 years of practice since the first national and international competition, respectively. In terms of performance level, there were 59 (35.75% of the sample) medal winners in the Paralympic Games or World Championships, and 106 (64.24%) participants competed at the Paralympic Games. The authors found that hours of training, the number of sessions, and the rate of perceived exertion (RPE) decreased significantly during the COVID-19-induced confinement. More specifically, hours of training decreased from 9.3 ± 4.3 to 5.5 ± 3.7, sessions reduced from 6.5 ± 3.1 to 4.6 ± 2.7 and RPE decreased from 5.0 ± 2.0 to 4.0 ± 2.0. Approximately 55% of the participants perceived that this reduction in training load along with the postponement of the Tokyo 2022 Paralympics Games would have been damaging and would have interfered with their careers. Particularly negative feelings were expressed by para-athletes of individual para-sports with opposition. This detrimental impact was greater among para-athletes with spinal cord injury and limb deficiency, when compared with other types of disability (visual, coordination, and peripheral impairment) and their able-bodied counterparts, and those taking part in team para-sports, when compared with individual sports.

Similarly, Urbański et al. (2021), conducting a questionnaire-based survey in Poland, recruited a sample of athletes with disabilities from 15 Paralympic sports disciplines, mainly suffering from spinal cord injury (paraplegia and tetraplegia, 25.9%), and limb amputation/deficiency (22.2%). The average time from diagnosis or injury was 20 ± 12.5 years, whereas the mean training experience was 10 ± 7 years. The authors found that most athletes with disabilities had to train at home (88.6%), while 60.2% of them could train outdoors. However, 12% of the interviewees had to suspend their training regimens, with only 5.4% of athletes with disabilities having some access to sports facilities and infrastructure. This resulted in a significant reduction in weekly training time (from 9.4 h/week *versus* 5.3 h/week, *p*-value <0.001). 60.8% of study participants reported difficulties and perceived barriers due to insufficient contact with assistants/caregivers or lack of access to assistive training devices. Finally, a significant majority of participants (more than 74%) were not satisfied with their training.

### Impact of COVID-19-induced confinement on mental health in para-athletes/athletes with disabilities

Eight studies have dealt with the impact of COVID-19 on mental health and psychological variables among para-athletes/athletes with disabilities (Clemente-Suárez et al., 2020; Dehghansai et al., 2021; Fiorilli et al., 2021; Hu et al., 2021; Kaneda et al., 2021; Kubosch et al., 2021; Martínez-Patiño et al., 2021; Busch et al., 2022). These populations have had to experience unique challenges during the COVID-19 pandemic, which has been “a tale of two stories”: the infectious outbreak and disability. Living with disabilities, these populations are at higher risk for contracting the emerging coronavirus compared with the general population, besides facing psychological distress (Busch et al., 2022; Dehghansai et al., 2021; Hu et al., 2021; Kaneda et al., 2021), due to several, intersecting mediators (Jesus et al., 2020; Lund, 2020; Lund et al., 2020; Jesus et al., 2021a; Jesus et al., 2021b; Kamalakannan et al., 2021; Goddard et al., 2022), as mentioned before. Dehghansai et al. (2021) performed a qualitative study interviewing seven Australian para-athletes, in order to capture the unique challenges of athletes with disabilities during the outbreak. The authors found high psychological distress levels among the participants who had to cope with their impairments during the pandemic, facing future uncertainty, budgetary constraints, and decentralized experiences. The athletes had to deploy two major strategies: namely, “anticipate and prepare” and “manage expectations”.

Hu et al. (2021) conducted a qualitative study (in-depth, semistructured interviews) and recruited a sample of para-athletes. They suffered from neurological disabilities (cerebral palsy, Wyburn-Mason syndrome, brain stem cerebellar injury, primary cerebellar degeneration, multiple sclerosis, spinal cord injury, spina bifida, transverse myelitis, and brachial plexus), limb deficiency (amputation, fibular hemimelia), visual disabilities (congenital glaucoma, retinitis pigmentosa, and other forms of visual impairment), and other complex, congenital syndromes (neuroblastoma, and Ehlers-Danlos syndrome). The authors found that the pandemic profoundly challenged their athletic identity, which is an important component of athletes’ self-concept and is closely related to health- and performance outcomes. More specifically, being highly challenged by the unprecedented situation, para-athletes had to re-structure their “Disability Sport Athletic Identity” (DSAI), and perceived the multiplicity rather than the exclusivity of their roles, to mentally adapt to the COVID-19-induced restriction and loss of physical participation in sport.

Kaneda et al. (2021) conducted a questionnaire-based, pre-post survey in Japan among a sample of 39 competitive athletes, including 20 para swimmers (8 females, 11 males) suffering from cerebral palsy, amputation, visual impairment, hemiplegia, spinal cord injury, osteoarthritis, and multiple sclerosis, and 19 able-bodied athletes. The authors found that female para-athletes reported worsening mental health, with higher apathy values than their able-bodied counterparts. Also, their male counterparts did not experience the same worsening mental health.

Kubosch et al. (2021) sampled the German paralympic athlete population. The authors found that 70% of the athletes felt that, during the COVID-19 pandemic, organizing their training was difficult, and two-thirds of the athletes trained less than before the pandemic period. Half of the participants worried about their own well-being, 25% about their career, and only 8% had economical-financial preoccupations. Similar results were reported by the same author group in another investigation (Busch et al., 2022), where seventy-eight paralympic athletes (40 women, 38 men, mean age 29.8 ± 11.4 years) were recruited and matched against the general population (mean age 30.5 ± 10.9 years). The para-athletes reported significantly lower scores on the “Patient Health Questionnaire 4” (PHQ-4) scale at each measurement time point compared to the matched control group. No age nor sex- and gender-specific effects could be detected.

Contrasting findings were obtained by Martínez-Patiño et al. (2021), who performed a cross-sectional, questionnaire-based survey in Spain and analyzed a sample of 64 Paralympic athletes (aged 28.4 ± 10.5 years old) *versus* 447 Olympic athletes (aged 26.0 ± 7.5 years), both nationally ranked or in the process of taking part into the Tokyo 2020 Olympic and Paralympic Games. The para-athletes were from the following disciplines: athletics, basketball, cycling, weightlifting, horse riding, judo, swimming, canoeing, taekwondo, tennis, table tennis, archery, Olympic shooting, and triathlon. The authors found that para-athletes were significantly differentially impacted by COVID-19 compared to their Olympic counterparts. More specifically, in the previous month, the former were more able to cope with their personal problems (r = 0.09, small effect size, *p*-value <0.010), reported fewer feelings of being lonely (r = 0.12, small effect size, *p*-value <0.047), and were more accepting of confinement measures (79.7% *versus* 59.9%). The authors suggested that the particular challenges and the unique psychological profile of the Paralympic athletes may have favored a psychological adaptation to the adverse situation. Furthermore, in another study conducted in Spain (Clemente-Suárez et al., 2020), recruiting 175 Olympic and 39 Paralympic athletes (aged 31.8 ± 9.3 years, competing in athletics, basketball, canoe, cycling, football, judo, swimming, table tennis, taekwondo, triathlon, weightlifting), para-athletes reported greater importance placed on the confinement, more perceived support from public institutions, and greater conscientiousness levels.

Finally, in line with Martínez-Patiño et al. (2021), Fiorilli et al. (2021) conducted a questionnaire-based, case-control study in Italy and recruited a sample of 146 athletes (73 without and 73 with disabilities, aged 42.11 ± 13.70 years). The authors found a lower subjective distress level among athletes with disabilities compared to their able-bodied counterparts (8.22% *versus* 30.14%, respectively). Overall, psychological distress was reported by 19.18% of the participants. Within the population of athletes with disabilities, age- and sports discipline-specific effects could be found with increasing age and individual sports participants displaying higher stress levels. No sex- and gender- or technical-level-specific effect could be computed.

### Impact of COVID-19-induced confinement on performance outcomes in para-athletes/athletes with disabilities

One study (Schipman et al., 2022) has investigated the impact of COVID-19-induced confinement on performance outcomes in para-athletes/athletes with disabilities. The authors conducted a data mining-based study, collecting data from 255 Paralympic events since 2010 and using the 4-year moving average, the slope of which was quantitatively assessed to evaluate changes in the temporal trend. The authors were able to find a dramatically detrimental and highly disrupting impact of COVID-19.

### Risk of contracting COVID-19 among para-athletes/athletes with disabilities

Only four studies assessed the risk of contracting the virus among para-athletes/athletes with disabilities and/or reported statistical figures on the COVID-19 rate in this specific athlete population (Urbański et al., 2021; Denerel and Lima, 2022; Kaneda et al., 2022; Muti et al., 2022). One of these investigations is a technical report on the feasibility of implementing a protocol aimed at protecting the health of paralympic athletes, named the “bubble scheme”, consisting of daily antigen testing, isolation, and avoidance of contact with the general public (Muti et al., 2022). The protocol was devised involving different skills and competencies, ranging from epidemiology and biostatistics, microbiology, sport and exercise medicine, and global and public health. The initial sample size of para-athletes was 23, with 12 followed up until the end of the study. The final para-rowing team consisted of 7 athletes, 3 coaches, and 2 team managers. No COVID-19 cases were found among this specific population: an aggressive testing strategy (552 polymerase chain reaction, PCR, tests, and 298 antigen-based tests) was carried out for an average number of 42 tests per athlete, showing that it is possible to create an “anti-COVID-19 protection bubble”, enabling para-athletes to compete in safe conditions. Slightly different results were found by Kaneda et al. (2022) and by Urbański et al. (2021). The former is a database-based epidemiological survey, in which the authors mined the website of the Japan Broadcasting Corporation NHK and the Tokyo Metropolitan Government. The COVID-19 infection rates in Tokyo during the Paralympic and Olympic Games were computed at 0.54% and 0.48%, respectively, while those among para-athletes and athletes were 0.30% and 0.25%, respectively. The infection rate was 1.13–1.20-times higher among para-athletes than among their able-bodied counterparts. In Poland, Urbański et al. (2021) reported that only 9% of the para-athletes of the study were quarantined and 1.8% of para-athletes and 0.6% of coaches contracted COVID-19. Finally, in Turkey, Denerel and Lima (2022) computed a slightly higher infection rate among able-bodied athletes (24.6%, n = 44) *versus* 23% (n = 42) among athletes with disabilities.

### Impact of COVID-19 infection on para-athletes/athletes with disabilities

Only one study (Denerel and Lima, 2022) investigated the impact of COVID-19 infection among para-athletes/athletes with disabilities. In particular, Denerel and Lima (2022) found that athletes with disabilities infected with SARS-CoV-2 displayed higher depression, anxiety, and coronavirus-related anxiety (CAS) scores than athletes with disabilities not infected with SARS-CoV-2. Sex- and gender-specific differences could be found, with females reporting higher scores. Athletes with disabilities competing in individual sports also reported higher scores than those participating in team sports.

## Discussion

### Impact of COVID-19-induced confinement on training and lifestyles in para-athletes/athletes with disabilities

The still ongoing COVID-19 pandemic has dramatically impacted athletes’ daily routines, by reducing training opportunities. This is particularly relevant for elite athletes, for whom regular training is highly recommended to preserve adequate muscular, and postural parameters (Alvurdu et al., 2022). As such, home-based training has been proposed as a valid strategy to counteract or, at least, partially mitigate against confinement-induced detraining, which can be further worsened by COVID-19-induced alterations (at the metabolic-respiratory, muscular, cardiac, and neurological levels) (Córdova-Martínez et al., 2022). However, athletes with disabilities and para-athletes may encounter difficulties in meeting with these recommendations, as they struggle more to train alone with respect to their able-bodied counterparts, and need consistent and appropriately trained help/personnel and specialized sports infrastructure (Cavaggioni et al., 2022).

Detraining has been studied among able-bodied athletes and is relatively overlooked among para-athletes/athletes with disabilities. There are a few studies that have explored detraining among people with disabilities (older adults aged ≥ 65 years with moderate mobility disability) in terms of alterations of mobility and gait biomechanics (Beijersbergen et al., 2016; Beijersbergen et al., 2017), showing changes in gait velocity, hip, ankle, and knee work.

### Impact of COVID-19-induced confinement on mental health in para-athletes/athletes with disabilities

The existing scholarly literature on the topic of athletes with disabilities and mental health has shown a higher burden in this specific population. For instance, Nabhan et al. (2021) found that, when compared with their able-bodied counterparts, athletes training for the Paralympics had a significantly higher percentage of positive screens for anxiety, depression, poor sleep quality, and sleep apnoea risk. Sport can have a buffering effect in terms of impact on mental health and well-being (Malm et al., 2019). It can promote resilience, improve emotional intelligence, strengthen other competencies, and increase self-esteem, self-perception, self-acceptance, and personal growth. All this significantly enhances the quality of life, and, more generally speaking, mental well-being. By means of sport, athletes with disabilities can build a “strong athletic *persona* (identity),” overcoming the social marginalization they can usually experience (Hu et al., 2021). Differential effects of type of sports discipline (individual versus team para-sport) were detected, suggesting that interactions and communications among peers can relieve and alleviate mental suffering, anxiety, and loneliness, enabling to react to adversities and challenging situations (Fiorilli et al., 2021). Moreover, athletes with disabilities can adopt particular coping strategies, such as avoidance, which is especially common among young para-athletes and athletes with disabilities, who are more likely to deny the implications and effects of COVID-19 and escape from negative emotions/feelings (Fiorilli et al., 2021). In conclusion, regarding confinement and mental health, a few of the studies included in the present scoping review found athletes with disabilities were better able to cope than athletes without an impairment, potentially due to the positive coping skills athletes with a disability may have (Martin, 2012; Dehghansai et al., 2021).

### Risk of contracting COVID-19 among para-athletes/athletes with disabilities

Exercise immunology among athletes with disabilities and para-athletes is a relatively underdeveloped and overlooked research field with respect to behavioral/environmental immunological studies conducted among their able-bodied counterparts. However, available scholarly evidence along with clinical experience shows that the unique underlying medical conditions in this specific population make them highly likely to develop an illness (Akashi et al., 2022). According to statistical figures, the incidence rate of illness is rather high in the Summer (10.0–13.2 episodes per 1,000 athlete-days) and Winter (18.7 episodes per 1,000 athlete-days) Paralympic Games (Janse Van Rensburg et al., 2018; Nieman and Wentz, 2019). Moreover, besides these underlying conditions, it is difficult for people with disabilities to adhere to and maintain strict precautions and public health measures of social distancing to avoid COVID-19 infection, especially among people with visual and motor limitations (Muti et al., 2022). Whereas one study did not find any COVID-19 cases among para-athletes/athletes with disabilities (Muti et al., 2022), other studies found an infection rate ranging from 0.3% to 1.8% (Urbański et al., 2021; Kaneda et al., 2022). The “bubble scheme” seems to be quite effective, even though a residual risk for COVID-19 remains, and is higher among para-athletes/athletes with disabilities than among able-bodied athletes or coaches/sports managers, being 1.13–3 time-higher (Urbański et al., 2021; Kaneda et al., 2022) in the Paralympic vs. Olympic Games. This suggests that more bespoke measures should be undertaken and interventions should be implemented to protect para-athletes/athletes with disabilities against COVID-19 like enhancing ventilation, providing free at-home testing kits, and improving tele-medicine, tele-rehabilitation, and other forms of remote support from healthcare workers and providers (Kaneda et al., 2022).

### Impact of COVID-19 infection on para-athletes/athletes with disabilities

The scholarly literature on the impact of COVID-19 infection on para-athletes/athletes with disabilities is very scarce. We were able, indeed, to retrieve only one relevant study. Moreover, to the best of our knowledge, data concerning the follow-up and the long-term effects of COVID-19 infection are lacking. A recently published systematic review found that approximately 94% of infected athletes displayed mild or no acute symptoms, with a small percentage of athletes experiencing persistent, long-term symptoms. COVID-19 is generally mostly mild in nature, but it can affect both return-to-play decisions and timing (Lemes et al., 2022). However, no data and recommendations could be given for athletes with disabilities, which warrants further research.

### Strengths and shortcomings of the present study

Our study presents several strengths, including the comprehensive literature search and appraisal, the methodological rigor, and the compliance with existing guidelines and checklists. On the other hand, it has also a number of limitations, that should be properly acknowledged. It was not possible to narrow our research question, given the limited number of available studies, so we preferred to broaden our research scope, also to give a broader overview and perspective on the topics under study. The heterogeneity of the literature and the contrasting findings reported preclude a more quantitative and meta-analytical approach.

### Critical considerations, implications, and future prospects

The scholarly literature is highly heterogeneous, with contrasting findings. Several hypotheses could be formulated to explain, at least partially, such discrepancies: differences in the methodology and study design (retrospective versus prospective, case-control with age- and sex-/gender-matched control subjects), country and stringency of the package of NPIs implemented, para-sport studied. Concerning the type of para-sport, some disciplines are unique, such as para-cycling, in that they require minimum outside equipment since there are minimal equipment needs and the equipment is available during the pandemic as it is personal equipment that is used outdoors. This could explain why Shaw et al. (2021) were not able to find any detrimental or disruptive effect of COVID-19 on training among para-athletes, since the outbreak could not influence outdoor activities and training, whereas Martínez-Patiño et al. (2021) and Clemente-Suárez et al. (2020) reported a positive response from Paralympic athletes to the COVID-19 pandemic. On the contrary, Cavaggioni et al. (2022), and Schipman et al. (2022) observed a negative impact of the infectious outbreak.

Differences among studies could also be due to different types of impairment evaluated, even though they are rarely reported: only in the studies by Cavaggioni et al. (2022), Hu et al. (2021), Trigo et al. (2022), and Urbański et al. (2021). Also, the composition/selection of the sample could have an effect. Trigo et al. (2022) noticed that paralympic athletes displayed more negative feelings and perceptions about the impact of COVID-19 on the training and performance outcomes when compared to their able-bodied counterparts. This could depend on the fact that, when the survey was conducted, most para-athletes recruited were still waiting for competition selection to the Paralympic Games and to take part in the international classification process. There was, as such, a great degree of uncertainty about the competition calendar, which translated and reflected in reduced load training. Other studies could have captured different times/moments of the international classification process, and, therefore, its effects in terms of expectations related to performance during the Tokyo 2020 Paralympic Games. The study country could have an impact too in terms of policies and public health intervention implemented: for example, in the investigation by Urbański et al. (2021), carried out in Poland, only 9% of athletes were quarantined and 1.8% of para-athletes and 0.6% of coaches contracted COVID-19.

Included studies exhibit some methodological limitations, like the small sample size, incidental/intentional opinion-type sampling for convenience, and unbalanced recruitment (with imbalance, for instance, in terms of para-sports disciplines), which limits the representativeness of the samples and hinders the generalization of the findings. Often, the design was correlational and univariate analyses were not adjusting for confounding variables, given the limited statistical power and the reduced small sample size. Furthermore, the response rate to the survey was in some cases low (ranging from 31.2–36% for the studies by Denerel and Lima, 2022, and by Clemente-Suárez et al., 2020, respectively, to 65.0% for the study by Busch et al., 2022), whereas in a few cases it was excellent (94% in the study by Urbański et al., 2021). Moreover, several measures have been retrospectively collected, which may lead to a recall bias and inaccuracy/unreliability of the results. Furthermore, a few studies, including Busch et al. (2022), did not properly compare the population of para-athletes/athletes with disabilities to a matched athlete population, but rather to the general population.

Furthermore, most studies did not correct for potential confounders, such as psychological variables (Urbański et al., 2021), nutritional uptake, or sleep quality (Trigo et al., 2022), which have been found to be altered during the COVID-19 pandemic and is known to impact the training schedules and performance outcomes among athletes. Finally, the use of self-designed instruments and tools makes it difficult to compare across different surveys.

### Recommendations for future studies

Based on our considerations, we recommend that standardized, reliable tools should be utilized and new, specific questionnaires should be created, tested for reliability, and validated. High-quality, multi-center, cross-countries, longitudinal surveys should be conducted to overcome all these shortcomings. Involving all relevant actors and stakeholders, including various national and international Paralympic Committees, as a few studies have done (Urbański et al., 2021) is fundamental: community-led, participatory research can help identify gaps in the current knowledge about sports-related practices among the population of athletes with disabilities during an unprecedented period of measures undertaken that have significantly affected everyday life. Moreover, this could advance the field, by capturing the needs of para-athletes and athletes with disabilities and enabling the design of a truly “disability-inclusive response” to COVID-19 and similar conditions/situations. Furthermore, follow-up studies on COVID-19-infected para-athletes and athletes with disabilities should be conducted. Evidence of long-term effects of COVID-19 is available only for able-bodied athletes, for whom cardiorespiratory residual alterations and mental health issues a long time after COVID-19 are described (Sala et al., 2020; Modica et al., 2022).

## Conclusion

Sport is known to play a key role for persons with disabilities, enhancing their socialization, improving their self-esteem, and increasing their quality of life and independence. Investigating the disruptive effect of COVID-19 is, therefore, essential to devise optimal strategies to counteract the negative effects of the infectious outbreak. However, despite the importance and relevance of this topic, evidence on the impact of COVID-19 on para-athletes is scant and contradictory, even though some discrepancies could be reconciled by taking into account the methodology adopted, the study design, the study country, and the type of para-sport assessed. All this calls for country-, para-sport-specific, data-driven recommendations. Athletes with disabilities and para-athletes have been affected to various extents by the COVID-19 pandemic, especially those who require access to specific training equipment and sports infrastructure, and those who take part in team sports who have lacked the social support of peers and other social networks, with subsequently reduced socializing opportunities. However, given the above-mentioned limitations and shortcomings, further research in the field is urgently warranted.

## Data Availability

The original contributions presented in the study are included in the article/supplementary material, further inquiries can be directed to the corresponding author.
